# A bibliometric analysis of linguistic research on COVID-19

**DOI:** 10.3389/fpsyg.2022.1005487

**Published:** 2022-09-13

**Authors:** Zhibin Peng, Zhiyong Hu

**Affiliations:** ^1^Foreign Language Research Department, Beijing Foreign Studies University, Beijing, China; ^2^Center for Linguistics, Literary and Cultural Studies, Sichuan International Studies University, Chongqing, China

**Keywords:** COVID-19, linguistics, bibliometric analysis, CiteSpace, hot topics

## Abstract

Research on COVID-19 has drawn the attention of scholars around the world since the outbreak of the pandemic. Several literature reviews of research topics and themes based on scientometric indicators or bibliometric analyses have already been conducted. However, topics and themes in linguistic-specific research on COVID-19 remain under-studied. With the help of the CiteSpace software, the present study reviewed linguistic research published in SSCI and A&HCI journals to address the identified gap in the literature. The overall performance of the documents was described and document co-citations, keyword co-occurrence, and keyword clusters were visualized *via* CiteSpace. The main topic areas identified in the reviewed studies ranged from the influences of COVID-19 on language education, and speech-language pathology to crisis communication. The results of the study indicate not only that COVID-19-related linguistic research is topically limited but also that insufficient attention has been accorded by linguistic researchers to Conceptual Metaphor Theory, Critical Discourse Analysis, Pragmatics, and Corpus-based discourse analysis in exploring pandemic discourses and texts.

## Introduction

The COVID-19 pandemic has impacted human beings in significant ways, and scientists and researchers have actively responded to the challenges in the post-pandemic era by investigating the phenomenon from the vantage point of their research domains. Since 2020, publications about COVID-19 have proliferated across disciplines. The COVID-19 research literature has also increased in bibliometric and scientometric studies (e.g., Chahrour et al., 2020; Deng et al., 2020; Colavizza et al., 2021), as well as systematic reviews and meta-analyses of a variety of COVID-19 pandemic-related topics, such as the risk factors for critical and fatal COVID-19 cases (Zheng et al., 2020) and considerations of whether asthmatic patients are at higher risk of contracting the virus (e.g., Morais-Almeida et al., 2020).

In response to the pandemic, linguistic researchers have provided multilingual public communication services or other helpful language services (Shen, 2020; Di Carlo et al., 2022). However, at this juncture, a clear need to map the contributions of the linguistic research community to pandemic literature was in evidence. Hence, the present study reviewed the COVID-19-related literature published in SSCI and A&HCI journals on the Web of Science over the past 2 years to address this need. The study used the CiteSpace bibliometric tool to analyze the current state of linguistic research on COVID-19. CiteSpace is a tool for performing a visual analytic examination of the academic literature of a discipline, a research field, or both, referred to as a knowledge domain (Chen, 2004, 2006, 2020). A bibliometric analysis is significant for recognizing the expansion of literature in linguistics. It can aid scholars in gaining quantitative insights into the rise of linguistic research on the COVID-19 pandemic, taking into account the social impact of the disease. The findings can identify the frontiers and gaps in the linguistic study on COVID-19 and guide future research.

## Previous studies

The COVID-19 pandemic has exercised a disruptive and profound impact on every aspect of human life. Scientific research papers concerning this pandemic have been growing exponentially. We searched publications related to this topic with “COVID” as the topic term in the Web of Science core collection and got 69,591 results^1^. To help researchers assess the research trends and topics on this issue, several literature surveys have already been implemented. Based on scientometric indicators or bibliometric analyses, these reviews include a focus on research patterns from publications on COVID-19 (Sahoo and Pandey, 2020), the most productive countries and the international scientific collaboration (Belli et al., 2020), and the current hotspots for the disease and future directions (Zyoud and Al-Jabi, 2020). The majority of these studies, however, have concentrated on the medical elements of COVID-19, while paying little attention to the research in the social sciences.

In this context, a recent review by Liu et al. (2022) based on a scientometric analysis of the performance of social science research on COVID-19, covering the landscape, research fields, and international collaborations, represents a notable departure from the prevalent focus of earlier studies. Representing a linguistic focus, another recent study by Heras-Pedrosa et al. (2022) consisted of a systemic analysis of publications in health communication and COVID-19. It found that, in 2020, concepts related to mental health, mass communication, misinformation, and communication risk were more frequently used, and in the succeeding year (2021), vaccination, infodemic, risk perception, social distancing, and telemedicine were the most prevalent keywords.

Within the linguistic field, literature reviews tend to focus on COVID-19-related language education exist. For instance, Moorhouse and Kohnke (2021) explore the lessons learned from COVID-19, and identify and analyze the primary knowledge produced by the English-language teaching community during the epidemic, also offering recommendations for further research on this particular subject. A systemic literature review of adult online learning during the pandemic by Lu et al. (2022) compiled and assessed 124 SSCI literature of empirical studies using a systematic literature review and the literature visualization tool CiteSpace. A bibliometric analysis on “E-learning in higher education in COVID-19” by Brika et al. (2022) deployed VOSviewer, CiteSpace, and KnowledgeMatrix Plus to extract networks and bibliometric indicators about keywords, authors, organizations, and countries. The study offered various insights related to higher education. Distance learning, interactive learning, online learning, virtual learning, computer-based learning, digital learning, and blended learning are among the many terms or subfields of e-learning in higher education.

Linguists have made notable contributions to COVID-19 research. However, there is currently no literature review available on the overall state of the field, including topics such as the most active contributors (e.g., countries, institutions, and journals) to research, dominant topic areas in the field, and trends and gaps in linguistic research. To bridge this gap, this study utilized CiteSpace software 6.1 R2 to conduct a systematic review of the present state of linguistic research on COVID-19. Specifically, this study addressed the following questions:

Q1: Which countries, institutions, and journals have contributed the most to the linguistic research on COVID-19?

Q2: What are the active research areas in the linguistic research on COVID-19?

Q3: What are the recent trends and the research gaps in the linguistic research on COVID-19?

## Methods

### Data collection

As the study was focused on the linguistic field, we searched the Social Science Citation Index (SSCI) and Arts and Humanities Citation Index (A&HCI) available on the Web of Science (WoS) platform. The data were collected through an advanced search. All collected articles/reviews were written in English, and we retrieved the data using the following fields:

1. Topic = (“covid*” OR “*nCoV” OR “SARS-CoV-2” OR “new coronavirus” OR “coronavirus disease 2019” OR “severe acute respiratory syndrome coronavirus-2” OR “novel coronavirus” OR “coronavirus 19”). These terms were only allowed in the title, abstract, or keywords.
2. Time span = 2020–2022
3. Document type = article OR review (the review articles do not include book reviews)
4. (“*”) is a wildcard in WOS that represents any group of characters, including no character.
5. Research area = “linguistics”

Based on the search items listed above, 363 research and review articles were obtained from the Web of Science Core Collection on 25 May 2022. Through manual analysis, the documents completely unrelated to linguistic research, as well as conference abstracts, book reviews, correspondence, and other unrelated documents were excluded. To guarantee the recall ratio, this study used the “remove duplicates (WOS)” function in CiteSpace to filter out duplicated studies from the collected data. After the cleaning procedure, the final dataset contained 355 documents.

### Instrument

The instrument deployed in this study was CiteSpace 6.1 R2 developed by Chen (2004) as a bibliometric analysis tool (Chen, 2004, 2006, 2017; Chen et al., 2010). The input in this software is a set of bibliographic data files in the field-tagged Institute for Scientific Information Export Format.

In this study, the files were downloaded from the WoS core collection. We chose “full record and cited references” as the record content and the files can be recognized by CiteSpace software directly. When the files are added to the software, they are subjected to the following procedural steps: time slicing, thresholding, modeling, pruning, merging, and mapping (For more details, please see Chen, 2004). The outputs of this software are visualized co-citation networks which is to say that each of the networks is presented in a separate interactive window interface. It can show the evolution of a knowledge field on a citation network, display the overall state of a certain field, and highlight some important documents in the development of a field. The strength of CiteSpace lies in the analysis and visualization of the thematic structures and research hotspots. It can provide us with co-citation networks among references, authors, and countries which is of pivotal importance given the research questions underpinning the present study. Hence, to locate important references, recognize research trends, and pinpoint research hotspots in the linguistic research on COVID-19, co-citation documents and keyword co-occurring analyses were conducted in this study through this software.

## Results

### Global distribution of articles on COVID-19

**Figure 1:**
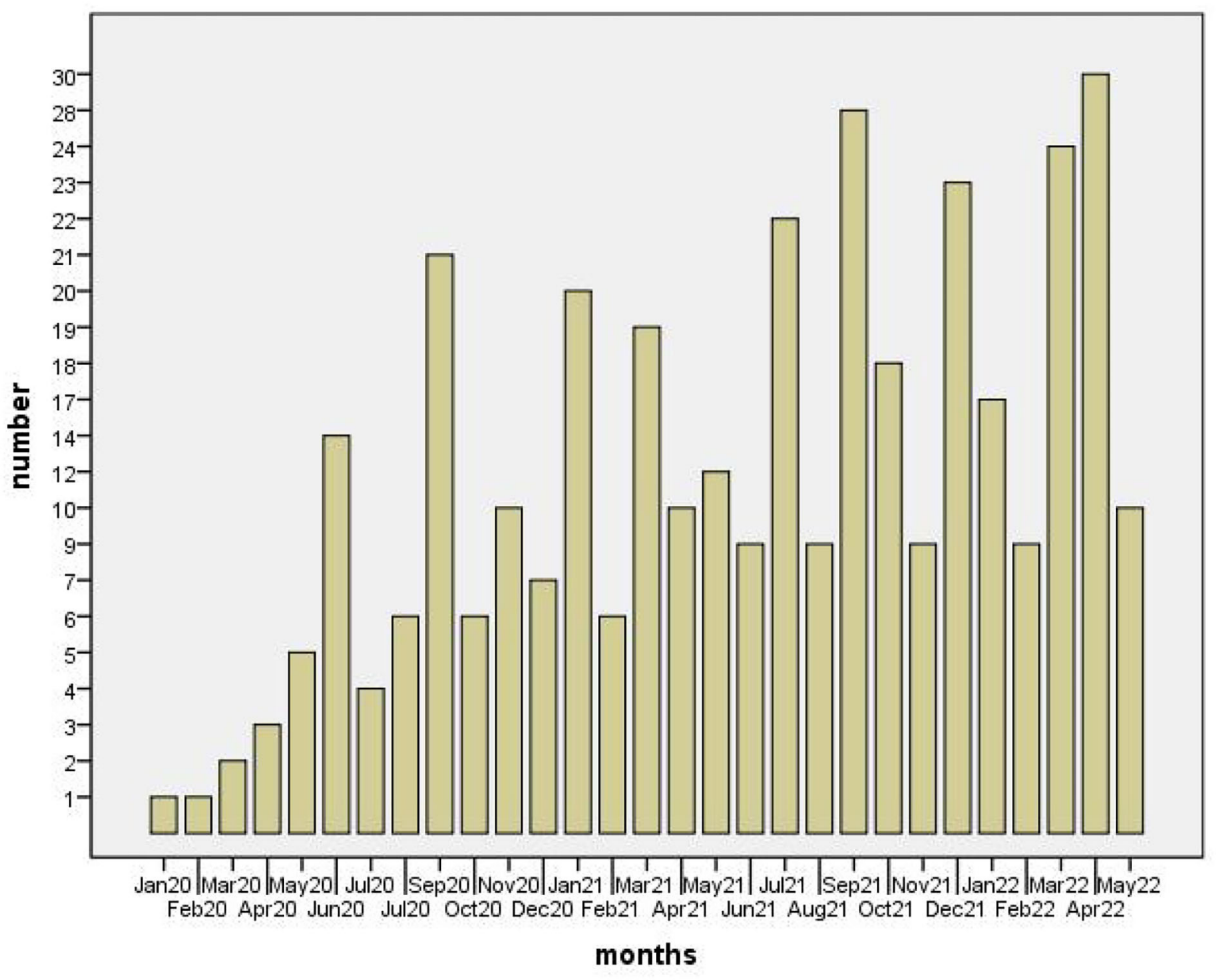
Number of articles published by month.

The overall distribution characteristics are presented below. Figure 1 displays the number of papers published each month since January 2020 when the World Health Organization formally declared the epidemic a global public health emergency. There was only one article about COVID-19 published in January 2020, whereas the publications show a peak in April 2022 with 30 publications. Overall, the results show that publications on the topic are increasing every month. Therefore, we might conclude that linguistic researchers have begun to be increasingly interested in COVID-19 linguistic research.

Tables 1, 2, respectively, indicate the top 10 most productive countries and institutions for COVID-19 publications. The USA was ranked as the top country in terms of the number of articles related to linguistic publications on COVID-19, with 111 publications in total, followed by China with 57 articles and England with 47 articles (Table 1). In terms of the number of linguistic research publications on COVID-19, Purdue University ranked as the top contributing institution (16 records), followed by the University of London (10 records) and the State University System of Florida (eight publications).

**Table 1:** Top 10 most productive countries for COVID-19 linguistic research.

**Ranking**	**Country**	**Records (Freq, total *N* = 355)**
1	USA	111 (31.3%)
2	China	57 (16.1%)
3	England	47 (13.2%)
4	Germany	32 (9.0%)
5	Australia	28 (7.9%)
6	Canada	19 (5.4%)
7	Spain	17 (4.8%)
8	New Zealand	10 (2.8%)
9	Austria	8 (2.2%)
10	Iran	7 (2.0%)
10	Italy	7 (2.0%)

**Table 2:** Top 10 most productive institutions for COVID-19 linguistic research.

**Ranking**	**Institutions**	**Records**
1	Purdue University	16
2	University of London	10
3	State University System of Florida	8
4	Michigan State University	7
4	University College London	6
6	Hong Kong Polytechnic University	5
7	Imperial College London	5
8	Northwestern University	5
9	Ohio State University	5
10	Adam Mickiewicz University	4
10	Columbia University	4
10	Education University of Hong Kong	4

The 355 articles reviewed in the current study were published in 83 journals. The top 10 most productive journals are listed in Table 3. *System* ranked the top journal in the number of published articles, with 21 publications related to COVID-19, followed by *American Journal of Speech Language Pathology* and *International Journal of Language Communication Disorders*, with 20 and 18 publications, respectively. As we can see in Table 3, most of the top 10 journals are related to language education or speech-language pathology.

**Table 3:** Top 10 most prolific journals.

**Ranking**	**Journal**	**Records**
1	System	21
2	American Journal of Speech Language Pathology	20
3	International Journal of Language Communication Disorders	18
4	Multilingual Journal of Cross Cultural and Interlanguage Communication	15
5	Foreign Language Annals	13
5	Linguistics Vanguard	13
5	RELC Journal	13
8	Language Assessment Quarterly	12
8	Metaphor and Symbol	12
10	Journal of Language and Social Psychology	11

Based on the Global Citation Score in the WoS, the top 10 most-cited articles contributing to COVID-19 research are listed in Table 4. MacIntyre et al. (2020) ranked as the most-cited article with 127 citations. This article is published in *System* which is also the most productive journal. The top four articles are all about online language teaching during the COVID-19 period.

**Table 4:** Top 10 most-cited articles contributing to COVID-19 linguistic research.

**Citation counts**	**Author (time)**	**Title**	**Journal**
127	MacIntyre et al. (2020)	Language teachers' coping strategies during the Covid-19 conversion to online teaching: Correlations with stress, wellbeing and negative emotions	System
52	Gacs et al. (2020)	Planned online language education vs. crisis-prompted online language teaching: lessons for the future.	Foreign Language Annals
39	Derakhshan et al. (2021)	Boredom in online classes in the Iranian EFL context: sources and solutions	System
38	Kohnke and Moorhouse (2022) (online time August 2020)	Facilitating synchronous online language learning through Zoom	RELC Journal
32	Piller et al. (2020)	Linguistic diversity in a time of crisis: Language challenges of the COVID-19 pandemic	Multilingua-Journal of Cross-cultural and Interlanguage Communication
28	Zaga et al. (2020)	Speech-language pathology guidance for tracheostomy during the COVID-19 pandemic: an international multidisciplinary perspective	American Journal of Speech-Language Pathology
22	Bischetti et al. (2021)	Funny but aversive: A large-scale survey of the emotional response to COVID-19 humor in the Italian population during the lockdown	Lingua
22	Marler and Ditton (2021)	“I'm smiling back at you”: Exploring the impact of mask wearing on communication	International Journal of Language and Communication Disorders
20	Russell (2020)	Language anxiety and the online learner	Foreign Language Annals
19	Alfadda and Mahdi (2021)	Measuring students' use of zoom application in language course based on the technology acceptance model (TAM)	Journal of Psycholinguistic Research

### Document co-citation analysis

**Figure 2:**
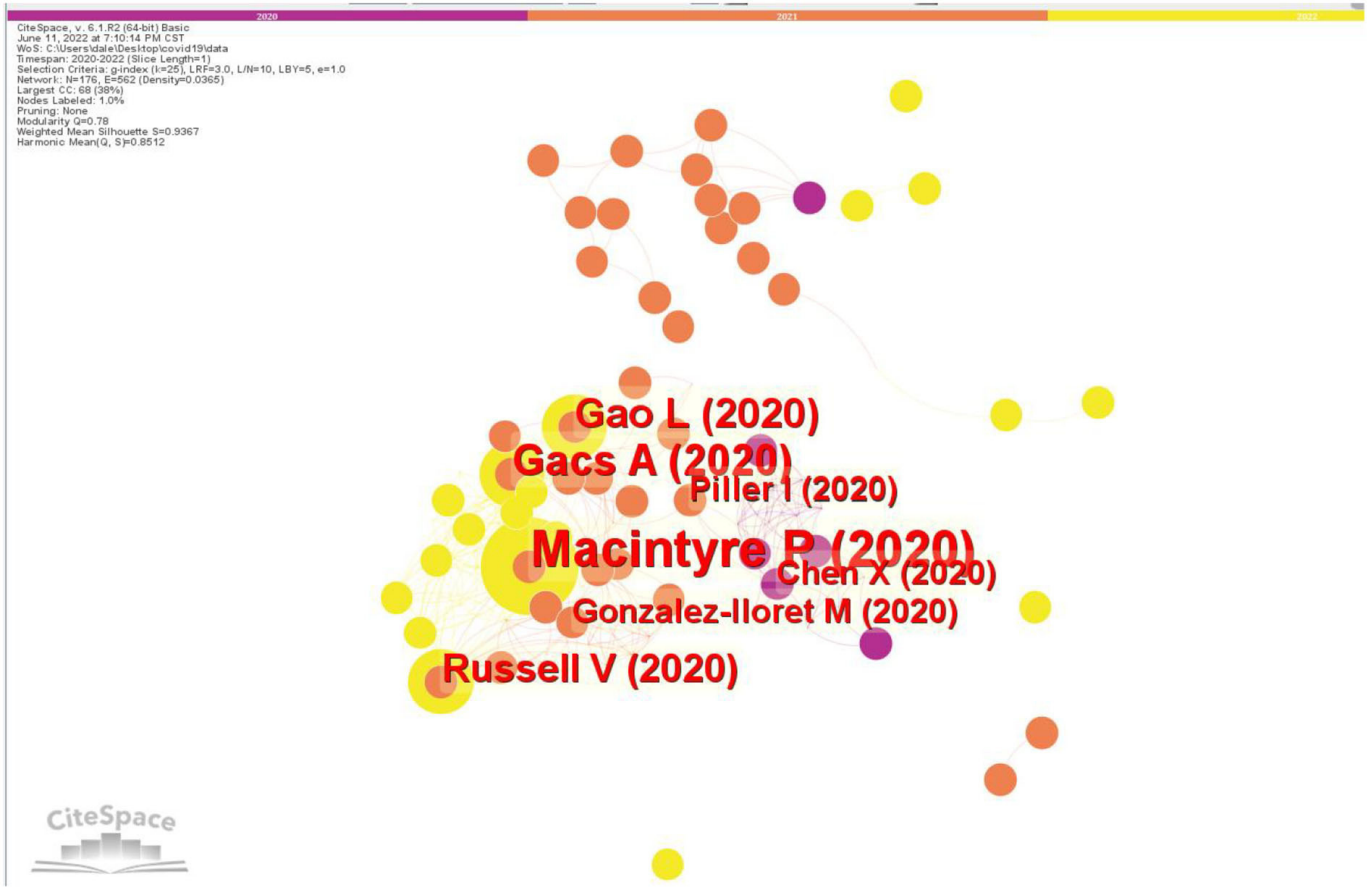
Critical articles in linguistic research on COVID-19.

The 355 bibliographic recordings from WoS were visualized and a 1-year time slice was selected for analysis. The size of the node is proportional to the frequency of the cited references. Different colors around nodes represent the frequency of references in different time periods. The labels shown in Figure 2 are all documents with more than three citations, and the connection between nodes shows the co-citation relationship.

The top 50 most cited articles every year were selected. There were 176 individual nodes and 562 links, representing cited articles and co-citation relationships among the whole data set, respectively. The results are illustrated in Figure 2. The results are somewhat different from those obtained from the Global Citation Score in the WoS (Table 4) since the Global Citation Score in the WoS is calculated based on all the citations in WoS, while the document co-citation analysis is based only on the 355 documents retrieved from WoS.

According to the document co-citation analysis, the most co-cited article was written by MacIntyre et al. (2020). This study explores the issue of language teachers' coping mechanisms and their correlates in the context of the distinctive stressors of the COVID-19 pandemic and the educational responses at the global level. It demonstrates how language teachers have faced a variety of challenges as a result of the global response to the COVID-19 outbreak. High levels of stress have been caused by the quick transition to online education, the blending of job and personal life, and the constant worry about personal and familial wellbeing. With the help of a variety of techniques, teachers were found to be dealing as effectively as they could. Coping strategies that are deemed to be more active and approach-oriented, namely ones that more directly addressed the problems brought on by the phenomenon including the emotions evoked, were found to be connected with more favorable outcomes in terms of psychological health and wellbeing. The greater use of avoidant coping mechanisms was linked to worse psychological outcomes. Increased use of avoidant coping, in particular, was linked to higher stress levels and a range of unpleasant feelings (anxiety, anger, sadness, and loneliness). MacIntyre et al. (2020) also found that a variety of particular techniques were employed by the participants within the approach and avoidant categories of coping, and the majority of them produced outcomes consistent with the category in which they appeared. The multidimensional nature of the stressors required multidimensional coping strategies, but it was obvious that some coping strategies were superior to others. This study by MacIntyre et al. (2020) offers insights into the effectiveness of coping strategies used by language teachers during the crisis and their implications for other stressful events and processes such as school transfers, educational reform, or demanding work periods like the end-of-year exam. MacIntyre et al. (2020) suggest that all pre-service and in-service teacher education programs should incorporate stress management as a fundamental professional competence.

The second most cited article is written by Gacs et al. (2020), which compares the crisis-prompted online language teaching during the COVID-19 era with well-designed and carefully planned online language education. Due to the 2020 pandemic, many institutions were forced to transition away from face-to-face (F2F) teaching to online instruction. The crisis-prompted online language teaching is different from actual planned online language education. This is because in times of pandemic, war, crisis, natural disaster, or extreme weather, neither teachers nor students are prepared for switching over to online education without good technology literacy, access, and infrastructure. Gacs et al. (2020) describe the process of preparing, designing, implementing, and evaluating online language education when adequate time is available and the concessions one has to make as well when adequate time is not a possibility in times of pandemic or in other emergent conditions. This article presents a roadmap for planning, implementing, and evaluating online education in an ideal and crisis contexts.

The third most cited article conducted by Gao and Zhang (2020) set up a qualitative inquiry to investigate how EFL teachers perceive online instruction in light of their disrupted lesson plans and how EFL teachers teach during the early-stage COVID-19 outbreak developed their information technology literacy. The findings from this study on teachers' perceptions of online instruction during COVID-19 have theoretical ramifications for studies on both teachers' cognitions and online EFL teaching.

It is evident that the three top-cited articles are on the theme of language education. Therefore, it can be concluded that remote online education during a pandemic crisis is the most studied area from the linguistic perspective.

### Keyword co-occurrence

In a way, keywords serve as the central summary of articles and serve to convey their major idea and subject matter. The co-occurrence of keywords in an article indicates the degree of closeness between the keywords and the strength of this relationship. According to common perception, the more strongly related two or more terms are, the more often they are likely to appear together. CiteSpace provides a function called Betweenness Centrality to describe the strength. In other words, if a keyword consistently appears alongside other distinct keywords, it is likely that we will see it even if we talk about other related subjects. As a result, the greater the value of Betweenness Centrality a keyword displays, the more significant a keyword is.

**Figure 3:**
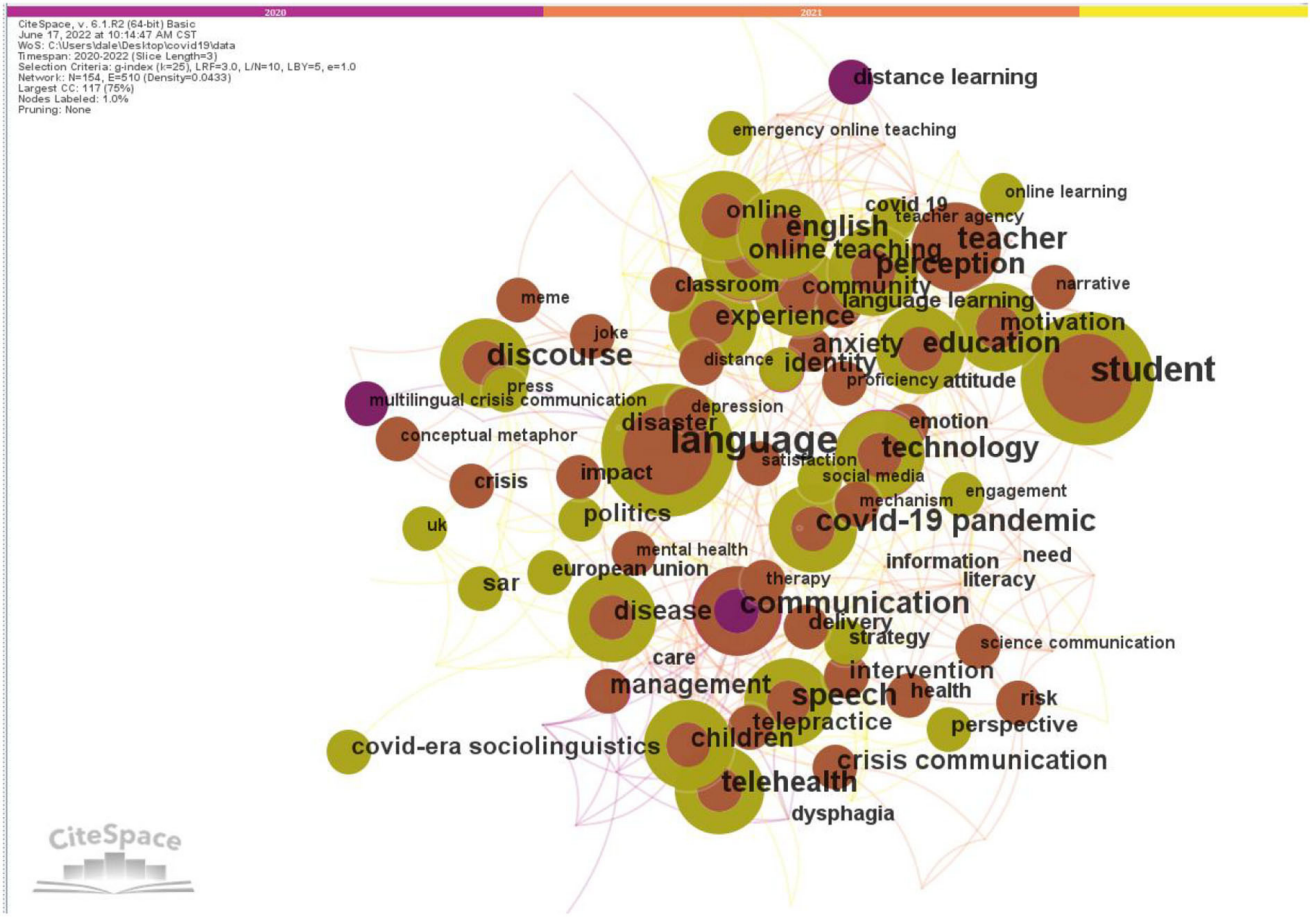
Keyword co-occurrence network for documents of linguistic research on COVID-19.

A keyword co-occurrence analysis was conducted in this study to identify the research fields and dominant topics. A term analysis of words extracted from keywords was conducted to identify the words or phrases co-occurring in at least two distinct articles. Terms with high frequency may be treated as indicators of hotspots in a certain research field (Chen, 2004). The top five high-frequency keywords were language, student, communication, discourse, and teacher. The keyword co-occurrence network is shown in Figure 3, and the keywords with frequencies of more than three are displayed in Table 5.

**Table 5:** The co-occurring keywords with high frequency.

**Count**	**Central**	**Keywords**	**Count**	**Central**	**Keywords**	**Count**	**Central**	**Keywords**
19	0.05	Language	6	0.04	Politics	4	0.01	Health
15	0.01	Student	6	0.03	Motivation	4	0.01	Strategy
11	0.18	Communication	6	0.03	Telepractice	3	0.05	Satisfaction
11	0.09	Discourse	6	0.01	Intervention	3	0.05	Therapy
11	0.02	Teacher	6	0.01	Sar	3	0.04	Distance
10	0.08	Speech	6	0.00	COVID-era sociolinguistics	3	0.03	Engagement
10	0.02	COVID-19 pandemic	5	0.09	Delivery	3	0.02	Meme
9	0.13	Technology	5	0.09	Distance learning	3	0.02	Press
9	0.10	Perception	5	0.03	Impact	3	0.01	Joke
9	0.09	Education	5	0.02	Language learning	3	0.01	Mental health
9	0.04	English	5	0.01	Perspective	3	0.01	Online learning
9	0.00	Telehealth	4	0.09	Care	3	0.01	Science communication
8	0.10	Online teaching	4	0.08	Literacy	3	0.01	Social media
8	0.06	Anxiety	4	0.06	Information	3	0.01	Teacher agency
8	0.03	Children	4	0.05	Classroom	3	0.00	Conceptual metaphor
7	0.12	Management	4	0.04	Need	3	0.00	Depression
7	0.10	Identity	4	0.04	Risk	3	0.00	Emergency online teaching
7	0.07	Experience	4	0.03	Attitude	3	0.00	Linguistic landscape
7	0.04	Disease	4	0.03	Emotion	3	0.00	Mechanism
7	0.02	Crisis communication	4	0.02	Crisis	3	0.00	Multilingual crisis communication
6	0.18	Community	4	0.02	Dysphagia	3	0.00	Narrative
6	0.10	Disaster	4	0.02	European union	3	0.00	Proficiency
6	0.05	Online	4	0.01	COVID-19			

### Cluster interpretations

**Figure 4:**
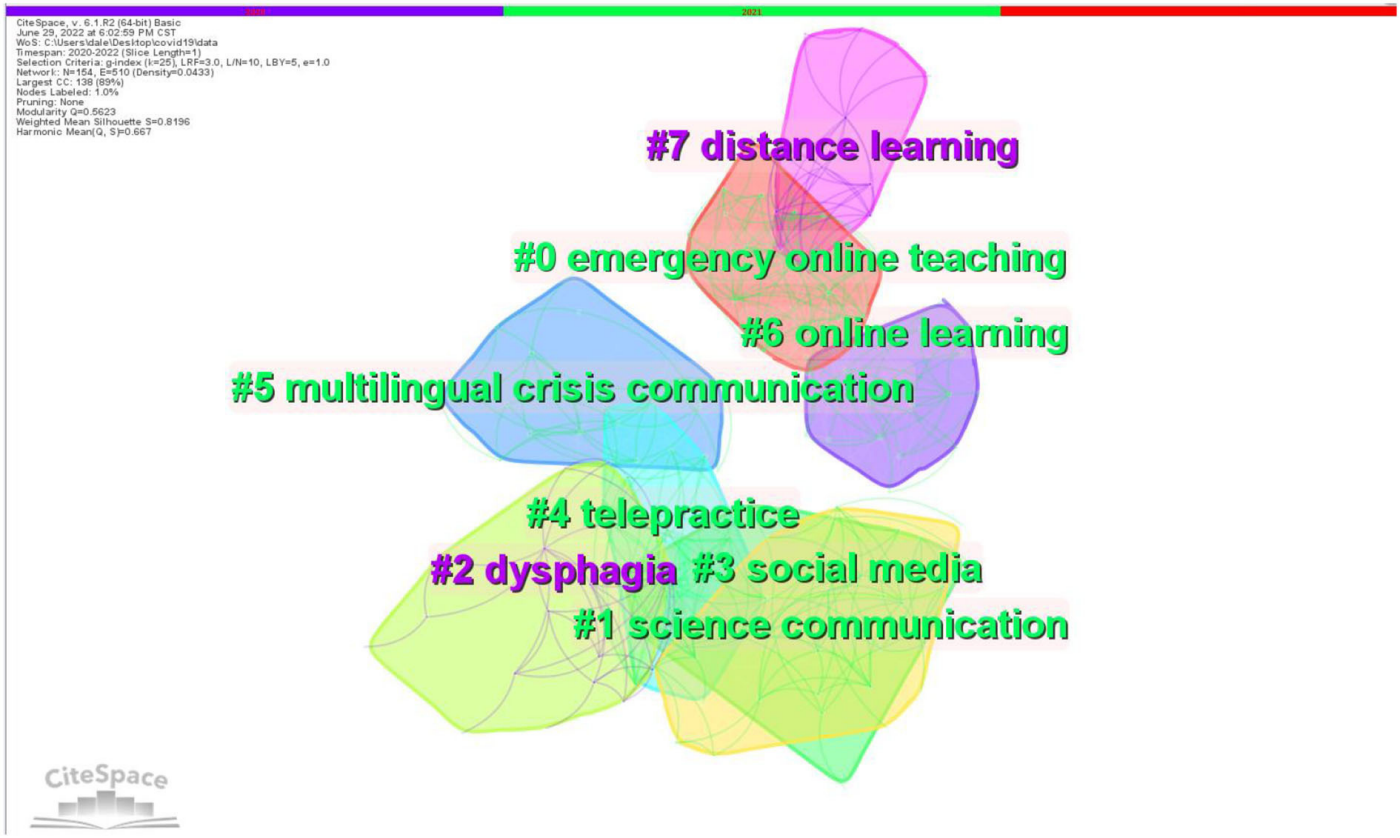
Cluster view of keyword co-occurrence.

Based on the analysis of the results of keyword co-occurrence, we used CiteSpace to conduct a cluster analysis. The 355 articles generated 20 clusters in total. Labeling clusters with indexing terms and showing clusters by log-likelihood ratio (LLR), Figure 4 shows the eight most important keyword clusters obtained by keyword co-occurrence analysis. Table 6 shows the keywords lists of the seven important clusters in linguistic research on COVID-19. It illustrates an aggregated distribution in which the most colorful areas overlapped, indicating that these clusters share some basic concepts or information (as suggested by Chen, 2004).

**Table 6:** Important clusters of keywords in linguistic research on COVID-19.

**Cluster ID**	**Size**	**Silhouette**	**Cluster names (LLR)**	**Top terms (LSI)**	**Top terms (LLR, p-level)**
#0	21	0.803	Emergence online teaching	Online teaching; emergency online teaching; teacher agency; critical incidents; New Zealand; online education; second language; reflective teaching; digital affordance; foreign languages	Emergency online teaching (11.03, 0.001); teacher agency (11.03, 0.001); online teaching (7.79, 0.01); autoethnography (7.33, 0.01); online education (7.33, 0.01)
#1	19	0.837	Science communication	Science communication; health psychology; corona virus; epistemic trust; COVID-19 discourse; time course; recovery; sentence comprehension; wh movement; agrammatic aphasia	Science communication (12.52, 0.001); hybrid instruction (4.14, 0.05); youth cultures (4.14, 0.05); information (4.14, 0.05); face-to-face instruction (4.14, 0.05)
#2	17	0.864	Dysphagia	Communication; aspiration pneumonia; utility; coordination; water reliability; scale; oropharyngeal dysphagia; validation; severity; dysphagia	Dysphagia (8.45, 0.005); outcm (8.45, 0.005); care (8.45, 0.005); patient (8.45, 0.005); COVID-19 (7.83, 0.01)
#3	17	0.814	Social media	European Union; critical discourse studies; European identities; media discourse; disaster linguistics; climate change; COVID-era sociolinguistics; COVID-19 pandemic; radical right	Social media (7.71, 0.01); European union (7.71, 0.01); climate change (7.71, 0.01); sar (7.71, 0.01); critical discourse studies (7.71, 0.01)
#4	17	0.816	Telepractice	Telepractice: intervention; children; experience; mental health; telehealth; perspective, speech; pathologist; ata practice	Telepractice (22.24, 1.0E-4); speech (13.72, 0.001); intervention (13.72, 0.001); telehealth (13.72, 0.001); children (9.11, 0.005)
#5	16	0.849	Multilingual crisis communication	Multilingual crisis communication; language challenges; intercultural dialogue; emergency linguistics; Chinese public health communication; remote teaching; foreign language teaching; post-secondary classrooms; prek-12 classrooms; covid	Multilingual crisis communication (7.49, 0.01); remote teaching (4, 0.05); pandemic (3.93, 0.05); dark humor (3.73, 0.1); context models (3.73, 0.1)
#6	16	0.675	Online learning	Online learning; foreign language; young learners; parental support; student experience; academic purposes; international students; remote teaching; pathway programs	Online learning (9.19, 0.005); teacher-student interaction (4.58, 0.05); pronouns (4.58, 0.05); social pain (4.58, 0.05); young learners (4.58, 0.05)
#7	12	0.882	Distance learning	Distance learning; online teaching; flexible learning; English language teaching; Philippine state university; teacher training; curriculum design; pre-service language teachers; flexible learning	Distance learning (30.76, 1.0E-4): English language teaching (12.05, 0.001); online teaching (12.05, 0.001); Philippine state university (12.05, 0.001); teacher training (12.05, 0.001)

#### Cluster #0 is labeled as emergence online teaching

Cluster #6 (online learning) and Cluster #7 (distance learning) are closely related to Cluster #0 since both Cluster #6 (online learning) and Cluster #7 (distance learning) fall under the umbrella of online education during a crisis. Emergency online teaching and online/distance learning are clearly shown to be the focus of linguistic research related to COVID-19.

Due to the COVID-19 pandemic, teaching and learning experienced a shift from physical, in-person (or face-to-face) learning environments to virtual, online learning environments. Although online education is well-established, pandemic-initiated online teaching and learning differed from traditional, well-planned online teaching, thus leading to significant difficulties for both language teachers and students. The stakeholders had to quickly adapt to new environments and learning styles while dealing with the pandemic's personal and societal repercussions on their everyday lives and wellbeing (MacIntyre et al., 2020). The online teaching of foreign and second languages during COVID-19 is referred to as emergency remote teaching (ERT), a term used to describe education temporarily moved online due to unforeseeable events such as natural catastrophes or conflict (Hodges et al., 2020). The difficulties primary school ESOL teachers in the United States encountered as a result of the unexpected instructional adjustments brought on by the COVID-19 epidemic are described by Wong et al. (2022) along with how these difficulties appeared to have impacted the teachers' wellbeing.

There are problems unique to language education, even if English language teachers and students have faced many of the same difficulties as their peers in other disciplines. For instance, many people view the interaction between students and teachers as a crucial component of language acquisition (Walsh, 2013), whereas interaction works very differently in the online mode (Payne, 2020). Therefore, to encourage and support engagement during online language lessons, teachers need to showcase certain competencies (Cheung, 2021; Moorhouse et al., 2021).

Understandably, the research community has developed a keen interest on how the COVID-19 pandemic has affected language teaching and learning. More attention is directed toward adapting to the COVID-19 pandemic-initiated online education due to the rapid and abrupt switch from classroom instruction to online learning. For instance, how the students—especially primary pupils—and the teachers adapt to online teaching is the main topic discussed in a special issue of *System* (2022, volume 105). The COVID-19 pandemic also changed the in-person and on-campus testing into placement testing. Ockey (2021) provides an overview of COVID-19's impact on English language university admissions and placement tests.

#### Cluster #1 is labeled as science communication

During the COVID-19 pandemic period, it has become very crucial for scientists and government politicians to communicate scientific knowledge to the public to limit the spread of COVID-19. Linguistic factors can play an important role in science communication. A study by Schnepf et al. (2021) inquired into whether complex (vs. simple) scientific statements on mask-wearing could lead audiences to distrust the information and its sources, thus obstructing compliance with behavioral measures communicated on evidence-based recommendations. The study found that text complexity affected audiences inclined toward conspiracy theories negatively. Schnepf et al. (2021) provided recommendations for persuading audiences with a high conspiracy mentality, a group known to be mistrustful of scientific evidence. Janssen et al. (2021) inquired into how the use of lexical hedges (LHs) impacted the trustworthiness ratings of communicators endeavoring to convey the efficacy of mandatory mask-wearing. The study found that scientists were perceived as being more competent and having greater integrity than politicians.

#### Cluster #2 is labeled as dysphagia

When a society faces a crisis like the COVID-19 pandemic, the impact of COVID-19 on special needs populations, such as people with dysphagia or aphasia or hearing impairments (Cheng and Cheng, 2022; Mathews et al., 2022), assumes greater importance for the linguistic community. A study by Jayes et al. (2022) described how UK Speech and language therapists (SLTs) supported differently abled individuals with communication disabilities to make decisions and participate in mental capacity assessments, best interest decision-making, and advance care planning during the COVID-19 pandemic. Govender et al. (2021) investigated how people with a total laryngectomy (PTL) were impacted by COVID-19. Feldhege et al. (2021) conducted an observational study on changes in language style and topics in an online Eating Disorder Community at the beginning of the COVID-19 pandemic. Owing to the severity of the pandemic, speech-language pathologists (SLPs) shifted quickly to virtual speech-language services. Thus, telepractice (cluster #4) also becomes one of the important keyword clusters. Telepractice has been used extensively to offer services to people with communication disorders since the global COVID-19 pandemic. Due to physical separation tactics used to contain the COVID-19 outbreak, many SLPs implemented a live, synchronous online distribution of clinical services. However, SLPs have received synchronous telepractice training to equip them for the shift from an in-person service delivery approach. Using synchronous modes of online clinical practice, Knickerbocker et al. (2021) provide an overview of potential causes of phonogenic voice issues among SLPs in telepractice and suggest prospective preventative techniques to maintain ideal vocal health and function.

Cluster #3 is labeled as social media and it is closely related to Cluster #5 (multilingual crisis communication) since social media research is a way to analyze public communication, particularly during a health crisis. Given the physical restrictions during COVID-19, social media platforms enabled individuals to maintain contact and share ideas. Many studies have investigated the performances of various types of social media platforms during the pandemic, such as Twitter (Weidner et al., 2021), Weibo (Ho, 2022; Yao and Bik Ngai, 2022), WhatsApp (Pérez-Sabater, 2021), and YouTube (Breazu and Machin, 2022). Weidner et al. (2021) looked at the characteristics of tweets concerning telepractice via the prism of a well-known framework for using health technology. During the epidemic, there was a surge in telepractice-related tweets. Although several tweets covered ground that is expected in the application of technology, some covered ground that might be particular to speech-language pathology. Yao and Bik Ngai (2022) investigated how People's Daily communicated COVID-19 messages on Weibo. Its findings contribute to the understanding of how public engagement on social media can be augmented via the use of attitudinal messages in health emergencies. Cluster #5 multilingual crisis communication is mostly studied from the perspective of sociolinguistics. Contributing to the sociolinguistics of crisis communication, Ahmad and Hillman (2021) examined the communication strategies employed by Qatar's government in dealing with the COVID-19 pandemic. While a study by Gallardo-Pauls (2021) proposed a specifically linguistic/discursive model of risk communication, Tu et al. (2021) inquired into how pronouns “we” and “you” affected the likelihood to stay at home differently. In another study, Tian et al. (2021) investigated the role of pronouns in crafting supportive messages and hope appeals and facilitating people to cope with COVID-19.

When a society is faced with a crisis, its language can reflect, reveal, and reinforce societal anarchy and divides. A study by Nagar (2021) examined how minority groups—Muslims and migrant workers—experienced marginalization, oppression, and damage through linguistic mechanisms such as silence, presuppositions, accommodations, othering, dog-whistling, and poverty.

## Implications for future study

As a discipline, linguistics has contributed significantly to the literature on COVID-19. Based on the results obtained from the above descriptive statistics and visualizations *via* Citespace, the study found that linguistic research on COVID-19 hitherto has largely focused on the influences of COVID-19 on language education, speech-language pathology, and crisis communication. Language education is one particular strand of applied linguistics, while speech-language pathology and crisis communication, respectively, comprise interdisciplinary studies of language and pathology, and language and communication.

The present state of linguistic research on COVID-19 reveals that there is a dearth of studies deploying linguistic theories such as Conceptual Metaphor Theory, Critical Discourse Analysis, Pragmatics, and Corpus-based discourse analysis. These theories can serve as important heuristics for exploring COVID-19 discourses. A strand of research from the perspective of these theories has highlighted the problematic nature of COVID-19 discourses.

Following the onset of the COVID-19 pandemic, linguists were concerned about the language regarding COVID-19. The Conceptual Metaphor Theory (Lakoff and Johnson, 1980), as one of the primary theoretical constructs in Cognitive Linguistics, was employed by some scholars to explore the COVID-19 discourse. Through their analysis of the conceptual metaphors in different kinds of COVID-19 discourse, linguistic scholars found that the WAR metaphor dominated the COVID-19 discourse (Bates, 2020; Chapman and Miller, 2020; Isaacs and Priesz, 2021). However, other metaphors such as FIRE remained underexplored concerning the pandemic (Semino, 2021). Although a study by Abdel-Raheem (2021) has explored the multimodal COVID-19 metaphor by examining political cartoons, in general, the multimodal COVID-19 metaphor has not been studied extensively. Further, despite the fact that Preux and Blanco (2021) experimental study explored the influence of the WAR and SPORT domains on emotions and thoughts during the COVID-19 era, the impact of the COVID-19 metaphor on the emotions and mental health of the public has received limited attention.

Critical Discourse Analysis has been deployed by some linguistic researchers. For example, critical discourse analysis was used by Zhang et al. (2021) to compare the reports on COVID-19 and social responsibility expressions in Chinese and American media sources. Based on a case study of U.S. regulations on travel restrictions during the COVID-19 pandemic, Li and Gong (2022) use proximization theory to demonstrate how proximization helps to legitimize health emergency measures. By using a multi-level content analysis technique based on theories of proximization and representation of distant suffering, Florea and Woelfel (2022) investigated the news portrayal of COVID-19 during the year 2020 as proximal vs. remote discourses of suffering. Forchtner and Özvatan, 2022 take a step toward the conceptual integration of narrative (genre) into the Discourse-Historical Approach in Critical Discourse Studies. Their study illuminated the far-right populist Alternative for Germany's (AfD) performances of delegitimization of itself/the nation in relation to Europe and legitimization of itself/the nation by articulating two paradigmatic, transnational crises: climate change and COVID-19. Szabó and Szabó (2022) used the discourse dynamics approach to identify the metaphorical terms employed by the Prime Minister to legitimize the crisis management of the Hungarian government and delegitimize critical commentary external to the European Union.

Drawing on critical discourse analysis and textual analysis, Zhou (2021) conducted an interdisciplinary study of the semiotic work dedicated to legitimating Traditional Chinese Medicine (TCM) treatment of COVID-19 in the social media account of an official TCM institution. While CDA analysis of COVID-19 discourses has been undertaken, more CDA-led studies need to be undertaken, given the complexity of power and inequities interwoven reflected in the texts and discourses pertaining to the pandemic.

Pragmatics research on COVID-19 is another underexplored area. Ogiermann and Bella (2021) analyze signs displayed on the doors of closed businesses in Athens and London during the first lockdown of the COVID-19 pandemic, providing some new insights into the dual function of expressive speech acts discussed in pragmatic theory. Blitvich (2022) explores the connections between face-threat and identity construction in the on/off line nexus by focusing on a stigmatized social identity (Goffman, 1963), a local ethnographically specific, cultural position (Bucholtz and Hall, 2005) attributed to some American women stereotypically middle-aged and white who are positioned by others as Karens. Thus, a woman who is perceived to be acting inappropriately, harshly, or in an entitled manner is categorized as a *Karen*. This incorrect behavior is frequently connected to alleged acts of racism toward minorities. The anti-masker *Karens* also achieved attention during the COVID-19 pandemic. This research offers a multimodal analysis of a sizable corpus, 256 films of persons whose actions and the way they were seen caused them to be positioned as *Karens*, to advance our knowledge of the Karen identity. More theories of Pragmatics, such as Relevance Theory, can be employed in the study of COVID-19 discourse.

Corpus-based COVID-19 discourse analysis is also deserving of research attention. Mark Davies has built the Coronavirus Corpus (https://www.english-corpora.org/corona/)—an online collection of news articles in English from around the world from January 2020 onwards. The corpus, which was first released in May 2020, currently has about 1,500 million words in size at the cutoff point (16 May 2022), and it continues to grow by three to four million words each day. It can provide vast original discourse data for researchers. For example, based on a 12.3-million-word corpus, Jiang and Hyland (2022) explore keyword nouns and verbs, and frequent noun phrases to understand the central concerns of the public reflected in its news media. In future, more research can be conducted based on the Coronavirus Corpus.

## Conclusion

Human life has been greatly affected and disrupted by the COVID-19 pandemic. Scientists and researchers have actively responded to this pandemic by investigating the phenomenon of COVID-19 from the lens offered by their fields of research, and publications relevant to COVID-19 have proliferated rapidly across disciplines since the beginning of 2020. To investigate contributions made by linguistic researchers to pandemic research, the current study carried out a bibliometric analysis of the relevant and available literature. Three hundred and fifty-five bibliometric recordings ranging from January 2020 to May 2022 were collected from WoS, and CiteSpace software was adopted to quantitatively and visually review these papers. The study found that there was continued growth in publications between January 2020 to May 2022. USA was found to be the most productive country in terms of contributions to literature contributing 111 publications pertaining to COVID-19, whereas *System* ranked as the top journal in the number of published articles related to COVID-19 (21 publications). Through the visualizations of keyword co-occurring analysis and cluster interpretation *via* Citespace, the study also found that linguistic research on COVID-19 focused largely on the influences of COVID-19 on language education, speech-language pathology, and crisis communication. However, the present review flags the need for more investigations of COVID-19 texts and discourses deploying the explanatory lens of key linguistics theories such as Conceptual Metaphor Theory, Critical Discourse Analysis, Pragmatics, and Corpus-based Discourse Analysis.

Although within its delineated scope, the present study aspired to be as comprehensive as possible, some limitations were unavoidable. For instance, the study searched documents in the Web of Science alone, not including other data sources such as Scopus, Google Scholar, Index Medicus, or Microsoft Academic Search. Further, only one scientometric tool was employed in this review. Future research may make use of a larger database and different analytical tools.

Nonetheless, this study comprises a pioneering review of linguistic research on COVID-19 and identifies and provides a clear overview of international linguistic research in relation to COVID-19. Hence, it can be used as a useful springboard by linguistic researchers interested in probing COVID-19 discourses and texts through the lens of leading theories in the field, thus not only expanding the topical breadth of linguistic research on the pandemic but also generating valuable insights in areas of pragmatics and metaphor as well as CDA and corpus research. These insights are likely to have theoretical as well as practical implications for the field of linguistics.

## Data availability statement

The raw data supporting the conclusions of this article will be made available by the authors, without undue reservation.

## Author contributions

All authors listed have made a substantial, direct, and intellectual contribution to the work and approved it for publication.

## Funding

This study was funded by the National Social Science Foundation Project (Award Number: 20XYY001, PI: ZH).

## Conflict of interest

The authors declare that the research was conducted in the absence of any commercial or financial relationships that could be construed as a potential conflict of interest.

## Publisher's note

All claims expressed in this article are solely those of the authors and do not necessarily represent those of their affiliated organizations, or those of the publisher, the editors and the reviewers. Any product that may be evaluated in this article, or claim that may be made by its manufacturer, is not guaranteed or endorsed by the publisher.

## Footnotes

^1^The search date is 15/05/2022.
